# Insights into intercontinental spread of Zika virus

**DOI:** 10.1371/journal.pone.0176710

**Published:** 2017-04-27

**Authors:** Dachao Liang, Ross Ka Kit Leung, Shui Shan Lee, Kai Man Kam

**Affiliations:** Stanley Ho Centre for Emerging Infectious Diseases, The Chinese University of Hong Kong, Hong Kong, China; National Cheng Kung University, TAIWAN

## Abstract

The epidemic of Zika virus (ZIKV) infection in South America has led to World Health Organization’s declaration of a Public Health Emergency of International Concern. To further inform effective public health policy, an understanding of ZIKV’s transmission mechanisms is crucial. To characterize the intercontinental transmission of ZIKV, we compiled and analyzed more than 250 gene sequences together with their sequence-related geographic and temporal information, sampled across 27 countries spanning from 1947 to 2016. After filtering and selecting appropriate sequences, extensive phylogenetic analyses were performed. Although phylogeographic reconstruction supported the transmission route of the virus in Africa, South-eastern Asia, Oceania and Latin America, we discovered that the Eastern Africa origin of ZIKV was disputable. On a molecular level, purifying selection was found to be largely responsible for the evolution of non-structural protein 5 and envelope protein E. Our dataset and ancestral sequences reconstruction analysis captured previously unidentified amino acid changes during evolution. Finally, based on the estimation of the time to the most recent common ancestors for the non-structural protein 5 gene, we hypothesized potential specific historic events that occurred in the 1940s and might have facilitated the spread of Zika virus from Africa to South-eastern Asia. Our findings provide new insights into the transmission characteristics of ZIKV, while further genetic and serologic studies are warranted to support the design of tailored prevention strategies.

## Introduction

Zika virus (ZIKV) is a mosquito-borne flavivirus, which was first isolated in 1947 in rhesus monkeys in Uganda [1]. Between 1950 to 1960, human infections were identified in Egypt, Nigeria, Uganda, India, Malaysia, Indonesia, Pakistan, Thailand, North Vietnam, and the Philippines [2]. Outside Africa and Asia, the first outbreak of ZIKV occurred on Yap Island in 2007[3]. In 2013, another wave of ZIKV infections spread to French Polynesia, the Cook Islands, New Caledonia and Easter Island [4,5]. In the Americas, the first confirmed case of ZIKV infection was reported in Brazil in May 2015 [6]. Since 2013, neurological disorders such as Guillain-Barré syndrome had been reported in outbreaks across French Polynesia and the Americas [7,8]. It was subsequently shown to be associated with an increased risk of microcephaly, and more recently with arthrogryposis [9]. In February 2016, the major ZIKV outbreak in Brazil was declared by World Health Organization (WHO) as a Public Health Emergency of International Concern (PHEIC). ZIKV then spread rapidly to other countries in South America, Central America and Caribbean. In the United States, the first case of local mosquito-borne ZIKV infectious was reported in November 2016 by the Texas Department of State Health Services [10]. As of February 2017, there were cumulatively over 200 000 autochthonous reported cases of Zika virus infection in the Americas. The epidemic has also found its way to Southeast Asia. In Singapore, following the first report of an first imported case in May 2016, a total of 115 ZIKV infections with 41 locally transmitted cases had been confirmed in three months [11].

Virologically, ZIKV consists of a single-stranded, positive-sense RNA with a genome of about 10.7kb in length. The genome encodes three structural proteins–capsid protein C (C), premembrane/membrane (prM), envelope protein E (ENV)–and 7 non-structural proteins (NS1, NS2A, NS2B, NS3, NS4A, NS4B, and NS5)[12,13]. A previous structural analysis proposed that a residue in ENV could possibly influence the transmission capacity of ZIKV[14]. Both cryo-electron microscopy and crystallization analyses supported the role of an asparagine residue as a glycosylation site for host cell attachment [14,15]. Envelope glycoproteins, capsid protein NS3 helicase and NS5 polymerase are the major targets of flaviviruses for antiviral agent development [16–18]. Using NS5 nucleotide sequences, ZIKV could divided into three major lineages: East Africa, West Africa and Asian[19]. Another research group took a different approach to the NS5 nucleotide sequences and classified ZIKV into two major lineages: African and Asian/American [20].

In studying ZIKV epidemiology, serologic analysis is cautioned because of concern of cross reactions, with the misdiagnosis of ZIKV infection as dengue [19]. Phylogenetic analysis [19]suggested that an outbreak strain collected in Yap in 2007 was closely related to the isolate from a monkey in Uganda in 1947[1]. A previous report indicated that the footprints of ZIKV had appeared in Africa, South and South-eastern Asia long before the recent outbreaks[2]. At least two reports had proposed East Africa as the origin of ZIKV [19,21]. Since ZIKV is transmitted mainly by the bite of *Aedes aegypti* mosquitoes [22], this explains the relative restriction of the distribution of the virus to tropical areas. More recently, ZIKV has been found to be transmitted by less common routes including vertically from mother to child [23,24], sexual contact [25] and blood transfusion [26,27]. To date, most studies had been based on the analyses of a small number of full genomes or a single gene, while very few studies [21,28] reported the recombination in those sequences. A recent study had included multiple genes but the focus was in structural analysis on adaptation [29].

There is clearly an urgent need to understand the overall intercontinental transmission pattern of ZIKV both in spatial and temporal context, which should be integrated with new knowledge on amino acid changes and the evolution of epidemic lineages. There is as yet no effective chemoprophylaxis, anti-viral treatment or vaccine to protect against ZIKV infection. The characterization and indepth analysis of ZIKV’s exact transmission patterns can provide evidence to inform effective public health policy in prevention and control of its spread. To this end, we collected a large number of ZIKV gene sequences and performed evolutionary and phylogeographical analyses. We proposed to divide ZIKV into lineages in accordance with the source of the sequence by continent, in an effort to understand the transmission dynamics of the virus over the last decades. In the course of the experiments, we discovered that the Eastern Africa origin of ZIKV was disputable. We also captured potential novel amino acid changes during evolution, as well as associated historical events that might facilitate the spread of Zika virus.

## Materials and methods

### Viral sequence retrieval and selection

Zika viral sequences covering the whole genome or polyprotein genes were retrieved from RefSeq (accessed on 1 March 2016). Coding regions of ENV, NS1, NS3 and NS5 were extracted from genome and polyprotein gene sequences. The four genes were also retrieved from Nucleotide database for individual gene based analysis. Sequences without collection date, location information, or of length shorter than 500bp were discarded. The numbers of ENV, NS1 and NS3 sequences included in our study were much smaller than that of NS5 (Table 1).

**Table 1:** Number of sequences per gene analyzed.

Gene	Number of sequences
ENV	56
NS1	33
NS3	31
NS5	131

To select appropriate gene for further analysis, all sequences were subjected to the following analysis. Multiple sequence alignment was performed by MAFFT v 7.273 with iterative refinement method incorporating local pairwise alignment information [30]. Recombination was assessed by the single breakpoint recombination (SBP) method [31] available on the Datamonkey web server [32] to ensure lack of recombinants in the gene segments for subsequent analyses. Maximum-likelihood trees were estimated using RAxML v 7.3.0 [33] with 1000 bootstraps under the GTR+Γ model. Reference genome sequences of Spondweni virus (NC_029055.1) was included as an outgroup. The most suitable nucleotide substitution model for the Bayesian Markov Chain Monte Carlo (MCMC) analysis was selected by bModeltest for all gene segment alignment datasets [34]. Demographic history of ZIKV was estimated using individual ENV, NS1, NS3 and NS5 gene and analyzed by Bayesian skyline plots [35]. NS5 is the longest coding region in the whole genome of ZIKV which plays an important role in RNA synthesis from the viral template [36]. For the estimation of genetic diversity over time, the NS5 dataset was also partitioned into two datasets representing sequences of African and South Pacific Rim lineages. The number, geographical coverage and time span of gene sequences were considered as criteria to select the gene for analyses, unless otherwise specified. Since the NS5 dataset consisted of the largest number of sequences with location and collection date information, this dataset was used in most analyses.

### tMRCA estimation

To explore the temporal scale of ZIKV evolution, nucleotide substitution rate and the time to the most recent common ancestors (tMRCAs) were estimated using a time-stamped Bayesian MCMC method as implemented in BEAST v2.3.2[37] using Coalescent Bayesian skyline model. Likelihood ratio tests were firstly performed to evaluate rate uniformity and the model of evolution among lineages by PAML [38] using default settings except that the TN93 substitution model [39] was employed. The maximum-likelihood tree produced in previous RAxML analysis was used as the starting tree in subsequent analyses. The relaxed clock model was then applied for further Bayesian MCMC method based analysis. For divergence time dating analysis, multiple calibration densities is needed [40]. Besides time-stamped sequences, two Yellow fever virus (YFV) sequences (Accession number: JF912184 and JF912181) were used for calibration, with a temporal prior of normal distribution with mean and standard deviation set to 305.5 and 77 years respectively, as described in a previous study [41]. Although historical records were used in calibration, potential uncertainties could still undermine its accuracy. We have therefore chosen the probabilistic prior distribution method, which has the benefit of eliminating uncertainties in calibration [42,43].

### Phylogeography

Nucleotide substitution rates, divergence times and demographic histories were estimated from the time-stamped ZIKV sequences using the Bayesian approach with the BEAST v2.3.2 package [37]. Discrete phylogeographic analysis was performed from these time- and location-stamped ZIKV sequences. Based on the United Nations geographical divisions, sequence location was defined as one of the eight regions, namely East Africa, West Africa, Middle Africa, South-eastern Asia, Oceania, South America, Caribbean and Central America[44]. We used 50 million Markov chain Monte Carlo (MCMC) chain length and discarded the first 10% as burn-in to generate 10 000 trees per run. Longer chain lengths were used if convergence was not achieved. An effective sampling size (ESS) higher than 200 was deemed as convergence. Maximum clade credibility trees were produced using TreeAnnotator v2.3.2 [37] with 1% burn-in. The two YFV sequences were also employed for calibration with the aforementioned parameters as well as the tMRCA estimation.

### Selective pressure analysis

We compared the results obtained by using PAML and Hyphy with FUBAR to investigate negative selection, BUSTED to underline the presence of diversifying selection, aBSREL to identify which branch was under diversifying selection, and MEME to find site-specific episodic of diversifying selection. During the PAML analysis, we also used the result obtained by M8, in addition to M0, M1, M2. To investigate selective pressure of different lineages in ENV and NS5, PAML [38] was first used to perform the analysis. Selective pressure of different lineages was estimated by two different categories of codon models, namely branch model, which computed the divergence of ratio of nonsynonymous and synonymous sites (dN/dS) for the five nodes (see Results) from the phylogeny; and sites model, which tested for specific sites under positive selection determined by Bayes empirical Bayes method [45]. Since codon substitution models M0 (one-ratio), M1 (nearly neutral), M2 (positive selection) and M8(βandω) were nested, LRT tests were performed to select the best-fit model for the data. With the default setting and using maximum-likelihood trees as the initial trees, models of fast unconstrained Bayesian AppRoximation (FUBAR)[46], mixed effects model evolution (MEME)[47], branch-site unrestricted statistical test for episodic diversification (BUSTED)[48] and adaptive Branch-Site Random Effects Likelihood (aBSREL)[49] in Hyphy package[50] were also used for conducting the parallel analyses. Since BUSTED[48] could be used to test both site-level and branch-level in gene-wild selection, the results of BUSTED[48] in site-level were compared with those from FUBAR[46], MEME[47] and PAML[38]. The selection result for branch-level from BUSTED[48] were compared with aBSREL[49]. In order to generate robust results, we only considered only if a site was reported under diversifying positive selection by at least 3 different methods. For a branch under diversifying positive selection, we considered only if it was reported under diversifying positive selection by two methods.

### Ancestral sequence inference and amino acid substitution analyses

We reconstructed the ancestral sequences of most recent common ancestors to the origin of ZIKV South Pacific Rim lineage, Pre-2007 and 2013 outbreaks and African lineage via maximum likelihood-based methods [51] available on the FastML web server [52] with the T92 substitution model [53] (the available model with parameters most similar to TN93) and gamma distribution. To validate the results, the analyses were also performed in PAML[38], HYPHY[50] and MEGA7.0[54] with the same dataset and TN93 substitution model.

## Ethics statement

This study had not involved the use of human or vertebrate animal subjects and/or tissue.

## Results

Following the collection of genome, polyprotein and individual gene sequences that contained the information of collection date and country, we obtained a total of 56 ENV, 33 NS1, 31 NS3 and 131 NS5 sequences, for this study. TN93 was the best fit model for all four genes by bModeltest analysis. Except for ENV, no significant (P<0.05) recombination was detected, implying that meaningful results from phylogeography and subsequent analysis can be developed. Breakpoint location was identified and removed at site 592 of the ENV sequences and we used only the first 591 nucleotides for subsequent analyses.

### Phylogeny and demographical history reconstruction

**Fig 1:**
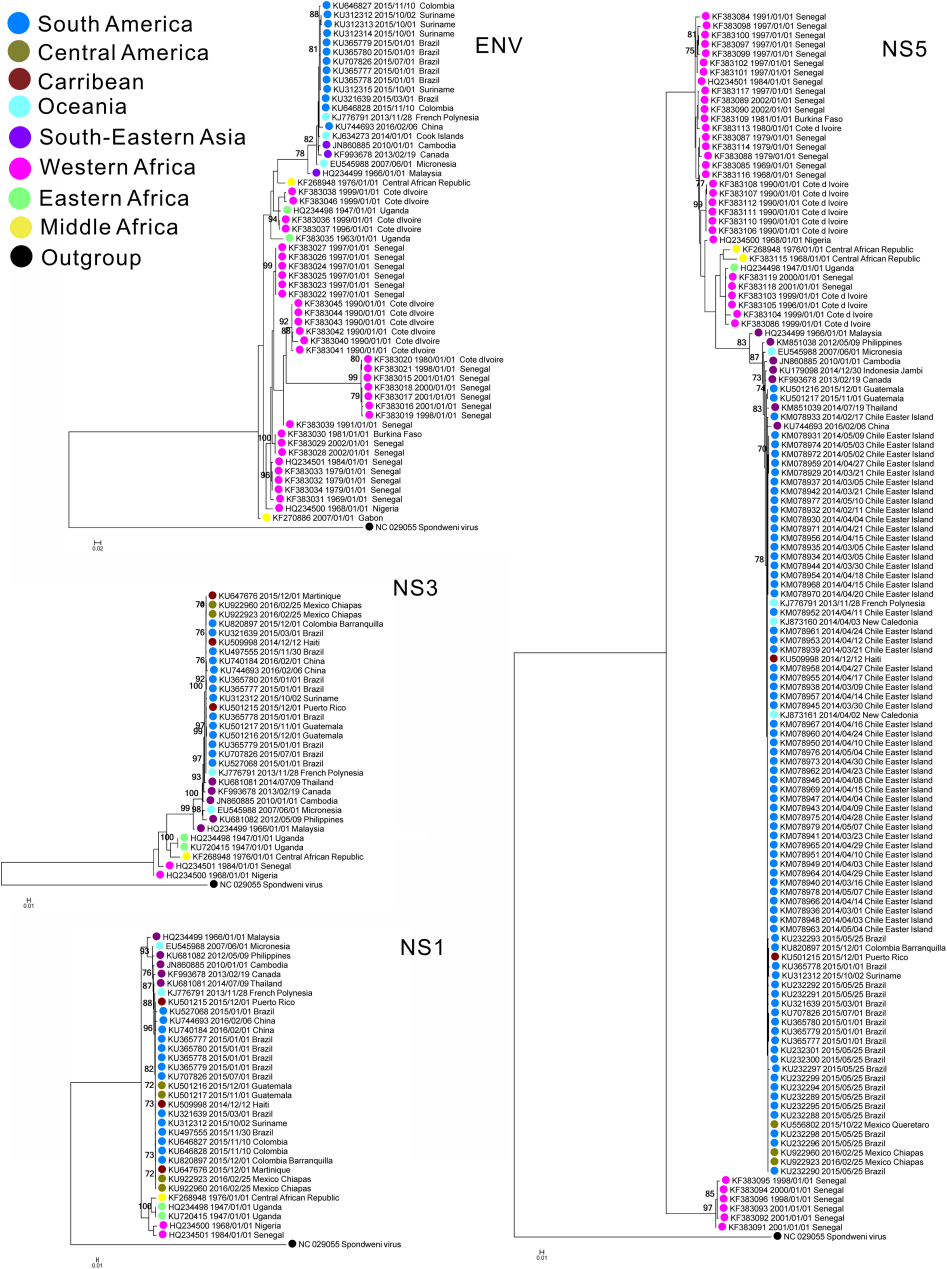
Maximum likelihood based phylogenetic tree with envelope protein E (ENV), nonstructural protein 1 (NS1), nonstructural protein 3 (NS3) and nonstructural protein 5 (NS5) sequences of the Zika virus (ZIKV). These trees are summarized after 1 000 replicates. Bootstrap values smaller than 70 are not shown. Since the evolutionary relationships of flaviviruses have been characterized[41], we used one of the closest evolutionary relationships species in flaviviruses, namely Spondweni virus (SPOV), to root the trees. The location for imported cases was assigned to the source country.

**Fig 2:**
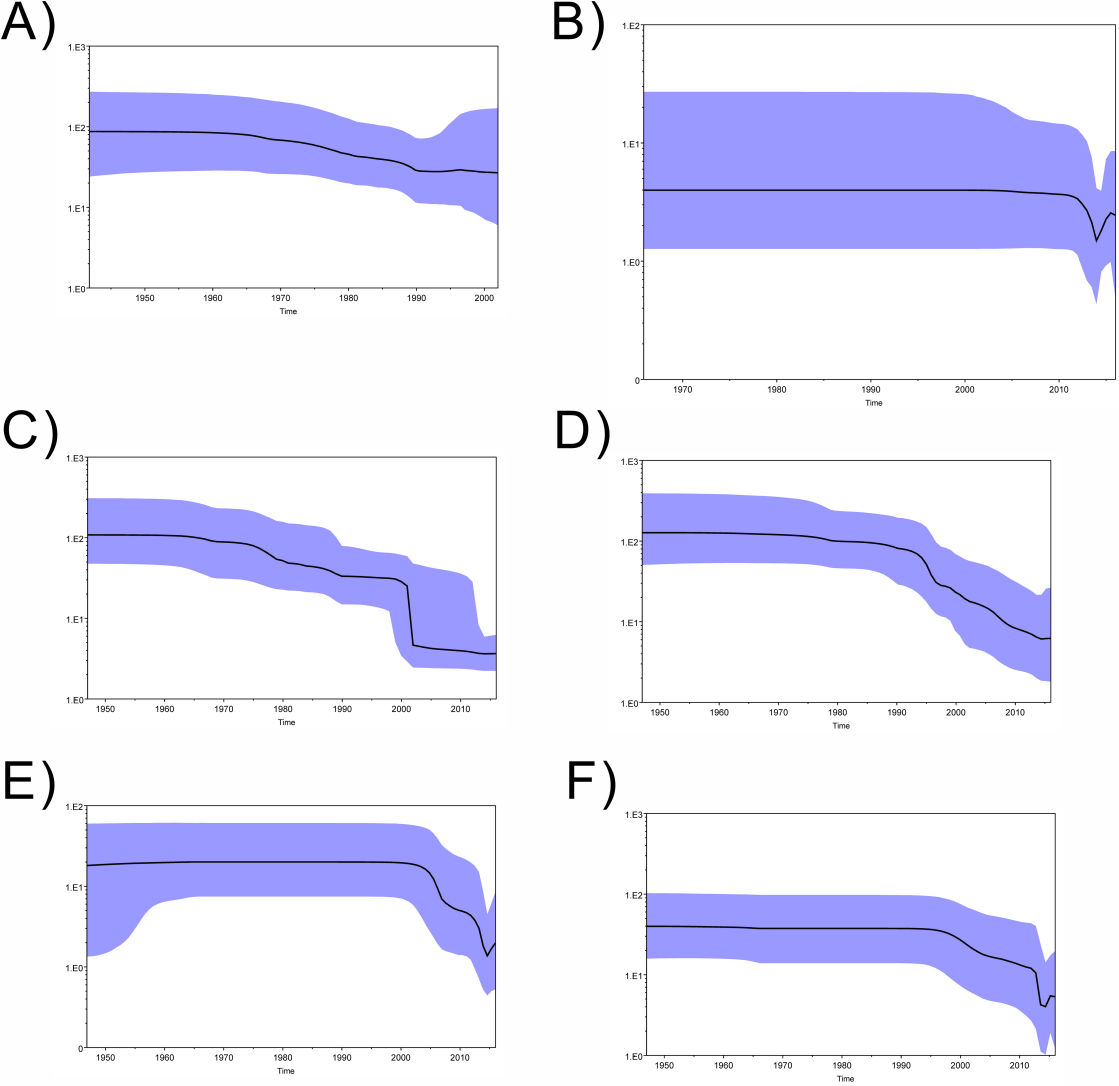
Zika virus (ZIKV) population dynamics of genetic diversity over time. The effective estimated population size of virus is shown on y-axis. X-axis shows the time before 2016. The colored area corresponds to the credibility interval based on 95% highest HPD. Mean and median values for relative genetic diversity (y-axis) together with credibility intervals were plotted through time (x-axis). (A) NS5 by African lineage (B) NS5 by South Pacific Rim lineage (C) NS5 (D) ENV (E) NS1 (F) NS3.

All four gene trees revealed two well-separated geographically distinct lineages of ZIKV, namely Asian and African, which had been identified in previous studies [3,19,55,56]. Western, Middle and Eastern Africa strains were monophyletic. The South-Eastern Asia strains occupied a basal position of the Asian lineage, whereas Oceania and Latin America strains were late-diverging (Fig 1). Higher genetic diversity was observed for the African lineage for all four genes and also seen in the NS5 Bayesian skyline plot analysis (Fig 2A and 2B). Notably, those pandemic strains collected after 2007 from Latin America and Oceania showed closer evolutionary relationships with Asian than African strains. The four gene trees also suggested a single introduction event of ZIKV to Latin America (Fig 1).

Bayesian skyline analysis from NS5, ENV, NS1 and NS3 genes all suggested a decrease in effective population size (N_e_) of Zika Virus (ZIKV) (Fig 2C–2F). It was estimated that N_e_ of ENV had dropped after 1990, ahead of other genes, though having the largest estimated extant N_e_ (about 10). The N_e_ of NS1 and NS3 showed a similar turning point of rising between 2013 and 2014, although the period was relatively short for a definitive conclusion to be made (Fig 2E and 2F). It is likely ZIKV outbreaks that occurred between 2013 and 2014 in Oceania [5,57] might account for this observation.

### Substitution rate estimation

LRT was performed by PAML[38] for considering suitable molecular clock model. The likelihood ratios results indicated that uncorrelated lognormal relaxed clock model (UCLN) was more probable and therefore it was chosen for use in subsequent analyses. Since strict molecular clocks could not be assumed for any of the four genes, subsequent analyses were based on the relaxed clock model. The estimated mean nucleotide substitution rates for NS1, NS3, NS5 and ENV were different, though their HPDs overlapped (Table 2). Previous estimates of ENV and NS5 substitution rates lie in the HPD of our study results [21] and there have been no previous estimates of substitution rates for NS1 and NS3.

**Table 2:** Mean nucleotide substitution rates with relaxed molecular clocks*.

Gene	Mean substitution rate (×10^−3^) (substitution/site/year)	Substitution rate HPD (×10^−3^) (substitution/site/year)
ENV	2.07	0.68–4.16
NS1	2.44	1.11–3.86
NS3	1.72	0.74–2.72
NS5	0.93	0.63–1.24

*LRT suggested that relaxed molecular clock model was more suitable compared to others.

### Estimation of geographical expansion

**Fig 3:**
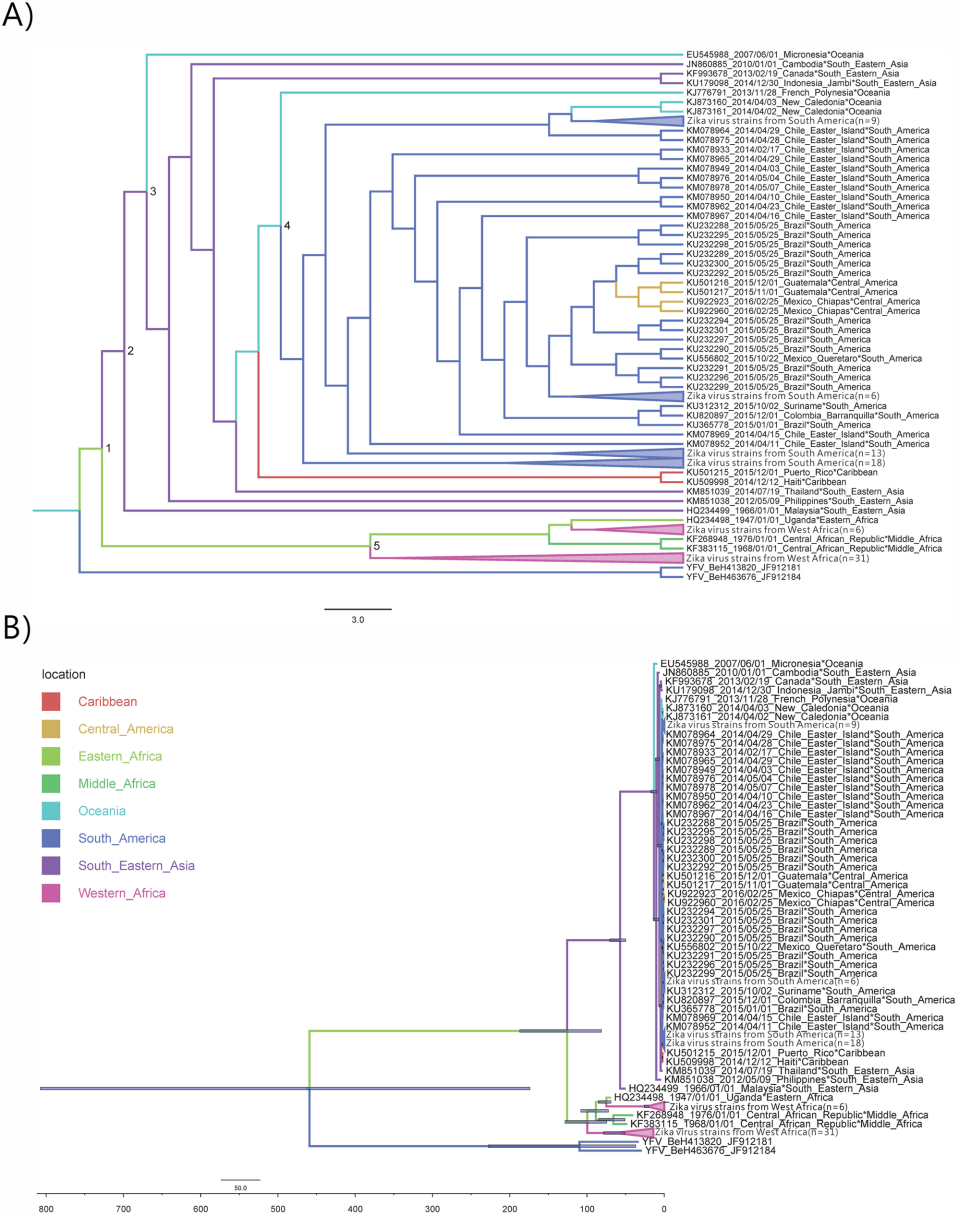
Bayesian phylogeographic tree of NS5. (A) cladogram, (B) phylogram, with the horizontal bar indicating the tMRCA 95% HPD of each node. Branches are colored according to the most probable location of the descendent nodes. Branches with the number of taxa fewer than five are collapsed for clarity. The time scale represents the number before the present time (2016-02-15).

Phylogeographic reconstruction suggested that the MRCA of ZIKV sampled in the last century (Fig 3A, Node 1) is disputable. Although Eastern Africa was still the most probable origin (S1 Table, probability = 24.70%), middle and western Africa were comparably likely (S1 Table, 19.36% and 16.43%), while the remaining locations also accounted for 8% each, totaling about 40%. The estimated year of appearance of this ancestral ZIKV was over a century ago (1887.26, 95% HPD interval: 1812.21.06–1932.98) (Fig 3B). This ancestor was predicted to have diverged into the African (Node 5) and South Pacific Rim (Node 2) lineages, with an apparent slowdown in the substitution rate in the former, though with overlap in 95% HPD intervals. The tMRCA of the Africa lineage was estimated to be 1914.78, with a rather wide 95% HPD interval (1876.85 to 1940.06). The South Pacific Rim ancestor, which eventually spread to South-eastern Asia, Oceania and South America, was estimated to appear only recently in 1947 (95% HPD interval:1941.35–1966.00). The pre-pandemic ancestor (Node 3) was estimated to first appear in the second half of 2002 (95% HPD interval: 1998.38 to 2006.24), possibly originating from South-eastern Asia (S1 Table, probability = 0.56) or Oceania (S1 Table, probability = 0.29) regions. Node 4 represents the tMRCA of the strains in the recent epidemic, which was estimated to be the second half of 2012 (95% HPD interval:2011.87–2013.00). The tMRCA for yellow fever virus (YFV)(JF912184 and JF912181) and ZIKV was estimated to be 1578.61, with 95% HPD interval as 1159.25 to 1845.89. The evidence therefore suggested the global ZIKV spread had originated in Africa, which was then transmitted to South-eastern Asia, Oceania, South America, Caribbean and Central America.

#### Amino acid substitution

To support the generation of hypotheses that can possibly explain recent outbreaks, we further examined inferred amino acid changes during the evolution. Consistent results obtained from using four different software platforms (FastML[52], PAML[38], HYPHY[50] and MEGA7.0[54]) are shown in Table 2. Six amino acid substitutions (K3061R, E3076K, V3085I, G3103R, K3157R and R3163S) were identified in the inferred ancestral NS5 sequences of ZIKV African lineage and the South Pacific Rim lineage. Half of the changes seemed to be less influential, including K3061R, V3085I and K3157R. Only three amino acid changes (R3103K, S3163N and S3219D) were found between the inferred ancestral sequences of South Pacific Rim lineage and Pre-2007/Pre-2013 outbreak strains (Table 3). The amino acid substitutions S3219D in NS5 have been reported between the selected pre-epidemic and epidemic strains in a previous study [58]. Ancestral sequence reconstruction, however, captured potential amino acid changes that could be missed by selected sequence analysis.

**Table 3:** Result of analyses on amino acid changes.

Polyprotein position	Sequence position	African lineage / Ancestor of ZIKV	South Pacific Rim lineage	2007/Pre-2013 outbreak strains
3061*	4	K	R	R
3076	19	E	K	K
3085	28	V	I	I
3103*	46	G	R	K
3157*	100	K	R	R
3163	106	R	S	N
3219	162	S	S	D

*Results that could be repeated by using three different software platforms.

#### Selection pressure analysis

Non-synonymous mutations had a low chance of being fixed (about 9% and 4% respectively for NS5 and ENV) in the population, as compared with a synonymous mutation by PAML[38]. Purifying selection signal was strong except for NS5 M1. According to LRT test results, M8 was the fittest model for the NS5 dataset. For ENV, however, M1, M2 and M8 were equally fit. We then determined positive selection sites by the Bayes empirical Bayes method. Only a single positively selected site in position 281 (numbering refers to accession number: YP_009227198.1, the origin position was 125) was identified for ENV. The Phe in position 281 was replaced by Ser. The dN/dS value of the site was estimated to be 1.649±0.838 (Probability = 0.899). Using the MEME[47] model by Hyphy[50], a total of 7 and 4 codons have shown detectable positive selection at significance level (P<0.05) in NS5 and ENV respectively. For codons in NS5, Site 23 was inferred to be under purifying selection 98% of the time, and under positive selection with 2% of the time. Sites 16, 105 and 138 were under positive selection 2–4% of the time while invariant in the remaining 96–98% of the time (ω = 0). Sites 26, 159 and 162 in consideration were under positive selection 4–18% of the time while invariant (ω = 0) in the remaining 82–96% of the time. For codons in ENV, site 55 was inferred to be under purifying selection 95% of the time, and under diversifying selection 5% of the time. Sites 17 and 182 were under positive selection around 3% of the time and invariant(ω = 0) in the remaining 97% of the time. Site 173 was under positive selection around 4% of the time while invariant (ω = 0) in the remaining 96% of the time. Only sites 23 and 138 were also reported under positive selection by BUSTED[48] at significant level (P<0.05) in NS5. In contrast, no site was notably under diversifying positive selection in NS5 and ENV by FUBAR[46] (posterior probability > = 0.9). The aBSREL model [49] was used to detect branches on which a proportion of codons evolved with ω > 1 at significant level (P<0.05). In the analysis, only the terminal branch “KF383104 1999/01/01 Cote d Ivoire” on NS5 had deviated from purifying selection to positive selection. But this result was not reported in the BUSTED model[48] applied. Table 4 summarized positive selection sites and branches by using different models in different software.

**Table 4:** Positive selection sites and branches under different models in PAML[38] and Hyphy[50].

Selection Model	Positive selection sites in NS5	Positive selection sites in ENV	Positive selection branches in NS5	Positive selection branches in ENV
PAML(M8)[38]	—	125	NA	NA
FUBAR[46]	—	—	NA	NA
MEME[47]	16,23,26,138,159,162	17,55,173,182	NA	NA
BUSTED[48]	23,138	—	—	—
aBSREL[49]	NA	NA	A terminal branch*	—

*The terminal branch was named “KF383104 1999/01/01 Cote d Ivoire”. NA: not applicable

## Discussion

In an effort to reconstruct the transmission history of ZIKV, we performed comprehensive sequence analyses by utilizing a large number of publicly accessible sequences, including their sequence-related spatial and temporal information, collected from 27 countries over the last few decades including time-points referable to known historical events. We inferred the demographic history of the ZIKV epidemic from Africa to the South Pacific Rim countries and estimated the associated evolutionary parameters.

In developing substitution model analysis, TN93 was the best-fit for all the four genes analyzed in this study. Our NS5 result was consistent with Frank et al. [59] except for ENV, but contrasted with Giovanetti et al.[29] who used the HKY model. Nevertheless, HKY differed from TN93[39] by unequal base frequencies assumption only, which could explain why our BSP results were similar. The BSP from all the four genes suggested a decrease in effective population size. However, no dependable positive selection signal was detected in the course of the evolution of the partial NS5 and ENV genes of ZIKV. Whether there were any adaptations in other genes, or any selective sweep or merely random genetic drift warrant further investigation.

Our study highlighted two uncertainties in determining the transmission history of ZIKV. One was the exact spatial origin of ZIKV, and the other was the probable association of 2013 Confederations Cup with the introduction of ZIKV to the South America. Although Eastern Africa was the most probable geographical origin, as previously suggested [21], western parts of Africa could be similarly likely as demonstrated in our results. Indeed, Africa only accounted for 60% of estimated probability. Faye et al estimated that the tMRCA of French Polynesian and America lineages was May 2013 (95% HPD: Dec 2012 to Sep 2013) [21]. Our estimation, however, suggested the tMRCA for the pre-2013 outbreak strains was unlikely to be later than the beginning of 2013 (2012.62 with 95% HPD from 2011.87 to 2013.00, Node 4 in Fig 3B). As a result, our result did not provide evidence to support the introduction of ZIKV around the time of the Confederations Cup, a sporting event held between May and June of 2013. This discrepancy could have arisen from the difference in both the number and sequence regions used for the estimation. Our predicted transmission pathway was similar to Chang’s and Giovanetti’s findings[29,60], that is, the global dissemination of ZIKV spread was likely to have originated from Africa, followed by eastward transmission to South-eastern Asia, Oceania, South America, Caribbean and Central America.

Relatively few studies on the origin of the South Pacific Rim lineage had been reported in the literature. The tMRCA for South Pacific Rim was estimated to be 1947 (95% HPD interval: 1941.35 to 1966) in our study. Coincidentally, during the Second World War in South-eastern Asia, around 100 000 East and West African soldiers were brought into combat in the Burma Campaign from January 1942 to July 1945 [61]. Specifically, the British Empire colonial unit 11th (East Africa) Infantry Division comprised troops from East and West African countries such as Kenya, Uganda, Nyasaland, Tanganyika and Rhodesia (Burma Star Association—The 11th East African Division). During those three-years’ conflict, both sides suffered heavy casualties, including at least 20 000 Japanese soldiers who died as a result of disease in the battle of Imphal [62]. It is also noteworthy that Thai army was also involved in this campaign and that after the Japanese surrendered, troops were continued to be deployed to the then Malaya. The whole campaign could serve as a possible portal of entry for the transmission of ZIKV from Africa to South-eastern Asia during that wartime period.

The recent ZIKV outbreaks in the South Pacific Rim could be a manifestation of serial founder effect. In perspective, the low genetic diversity of the South Pacific Rim lineage, speculated migrations over long distances, and the routes through islands with isolated populations could have affected the space-time dynamics of virus transmission. Notwithstanding the few potential amino acid changes observed during the evolution, no definitive positive selection signal was detected for NS5. Only one of the two important structural sites Phe279Ser and Val311Ile in ENV reported by Giovanetti et al [29] was identified as a positive selection site. Our dN/dS estimates for NS5 and ENV reinforced the view that synonymous mutation accumulation and purging of deleterious polymorphisms were most probable during ZIKV transmission [21,29]. Protein structure modeling and analysis may provide clues to evaluate whether the reported amino acid changes can have any functional consequences. Finally, as immunity to ZIKV can be context dependent [19], genetic and serologic comparison of the outbreak strains with the more “primitive” strains, which may have been endemic in parts of Africa and India [63], would be necessary in order to devise specific vaccination strategies for future control of the epidemic.

One major limitation of our study was that we had not addressed the possible association of genetic differences with variability in clinical presentation or the neuro-tropism of different virus strains amongst infected populations. There could well be host genetic factors which could influence our phylogeographic findings. Bearing in mind the already extensive spread of other vector borne viruses (e.g. dengue), it seems likely that ZIKV would follow the same tracks. In particular, population events (e.g. war time events, mass refugee movements) may further enhance its rapid spread. In future, evidence can be collected by comparing similar vector borne viruses, so that better knowledge can be gained in understanding their possible mechanism of spread amongst human populations.

## Supporting information

S1 Table Probability value of individual nodes. (DOC)

S2 Table Accession number of sequences included in this study. (DOC)
