# Supplementary material for: Insights into intercontinental spread of Zika virus
Source: PLoS One. 2017 Apr 27;12(4):e0176710. doi: 10.1371/journal.pone.0176710 (PMC5407806; doi:10.1371/journal.pone.0176710)
Supplement: S1 Table — (DOC) [file pone.0176710.s001.doc]

**S1 Table. Probability value of individual nodes**

| Node | 1 | 2 | 3 | 4 | 5 |
| --- | --- | --- | --- | --- | --- |
| Representation | MRCA of ZIKV collected in this study | South pacific rim lineage | 2007 outbreak lineage | Pre-2013 outbreak lineage | African lineage |
| Western Africa | 0.1643 | 0.0047 | 0.0003 | NA | 0.2038 |
| Eastern Africa | 0.2470 | 0.0339 | 0.0148 | 0.0026 | 0.3472 |
| Middle Africa | 0.1936 | 0.0284 | 0.0105 | 0.0017 | 0.2333 |
| South-Eastern Asia | 0.0811 | 0.5949 | 0.5637 | 0.0315 | 0.033 |
| Oceania | 0.0866 | 0.1711 | 0.2901 | 0.8303 | 0.0416 |
| South America | 0.0733 | 0.0397 | 0.026 | 0.0932 | 0.0383 |
| Central America | 0.0657 | 0.0278 | 0.0118 | 0.0148 | 0.0454 |
| Caribbean | 0.0886 | 0.0994 | 0.0827 | 0.0258 | 0.0573 |
| Posterior probability | 0.9994 | 1.0000 | 1.0000 | 1.0000 | 0.5714 |
