# Supplementary material for: Insights into intercontinental spread of Zika virus
Source: PLoS One. 2017 Apr 27;12(4):e0176710. doi: 10.1371/journal.pone.0176710 (PMC5407806; doi:10.1371/journal.pone.0176710)
Supplement: S2 Table — (DOC) [file pone.0176710.s002.doc]

**S2 Table. Accession numbers of sequences included in this study**

| Virus Name | Accession number | Country |
| --- | --- | --- |
| ZIKV | KU922923 | Mexico |
| ZIKV | KU922960 | Mexico |
| ZIKV | KU820897 | Colombia |
| ZIKV | KU179098 | Indonesia |
| ZIKV | KU707826 | Brazil |
| ZIKV | KU232288 | Brazil |
| ZIKV | KU232289 | Brazil |
| ZIKV | KU232290 | Brazil |
| ZIKV | KU232291 | Brazil |
| ZIKV | KU232292 | Brazil |
| ZIKV | KU232293 | Brazil |
| ZIKV | KU232294 | Brazil |
| ZIKV | KU232295 | Brazil |
| ZIKV | KU232298 | Brazil |
| ZIKV | KU232297 | Brazil |
| ZIKV | KU232296 | Brazil |
| ZIKV | KU232299 | Brazil |
| ZIKV | KU232300 | Brazil |
| ZIKV | KU232301 | Brazil |
| ZIKV | KU509998 | Haiti |
| ZIKV | KU556802 | Mexico |
| ZIKV | KU501215 | Puerto Rico |
| ZIKV | KU501216 | Guatemala |
| ZIKV | KU501217 | Guatemala |
| ZIKV | KU365777 | Brazil |
| ZIKV | KU365778 | Brazil |
| ZIKV | KM078931 | Chile |
| ZIKV | KM078930 | Chile |
| ZIKV | KM078929 | Chile |
| ZIKV | KM851038 | Philippines |
| ZIKV | KM851039 | Thailand |
| ZIKV | KU312312 | Suriname |
| ZIKV | KU365779 | Brazil |
| ZIKV | KU365780 | Brazil |
| ZIKV | KU744693 | China |
| ZIKV | KU321639 | Brazil |
| ZIKV | KM078932 | Chile |
| ZIKV | KM078933 | Chile |
| ZIKV | KM078934 | Chile |
| ZIKV | KM078935 | Chile |
| ZIKV | KM078936 | Chile |
| ZIKV | KM078937 | Chile |
| ZIKV | KM078938 | Chile |
| ZIKV | KM078939 | Chile |
| ZIKV | KM078940 | Chile |
| ZIKV | KM078941 | Chile |
| ZIKV | KM078942 | Chile |
| ZIKV | KM078943 | Chile |
| ZIKV | KM078944 | Chile |
| ZIKV | KM078945 | Chile |
| ZIKV | KM078946 | Chile |
| ZIKV | KM078947 | Chile |
| ZIKV | KM078948 | Chile |
| ZIKV | KM078949 | Chile |
| ZIKV | KM078951 | Chile |
| ZIKV | KM078950 | Chile |
| ZIKV | KM078952 | Chile |
| ZIKV | KM078953 | Chile |
| ZIKV | KM078954 | Chile |
| ZIKV | KM078955 | Chile |
| ZIKV | KM078956 | Chile |
| ZIKV | KM078957 | Chile |
| ZIKV | KM078958 | Chile |
| ZIKV | KM078959 | Chile |
| ZIKV | KM078960 | Chile |
| ZIKV | KM078961 | Chile |
| ZIKV | KM078962 | Chile |
| ZIKV | KM078963 | Chile |
| ZIKV | KM078964 | Chile |
| ZIKV | KM078965 | Chile |
| ZIKV | KM078966 | Chile |
| ZIKV | KM078967 | Chile |
| ZIKV | KM078968 | Chile |
| ZIKV | KM078969 | Chile |
| ZIKV | KM078970 | Chile |
| ZIKV | KM078971 | Chile |
| ZIKV | KM078972 | Chile |
| ZIKV | KM078973 | Chile |
| ZIKV | KM078974 | Chile |
| ZIKV | KM078975 | Chile |
| ZIKV | KM078976 | Chile |
| ZIKV | KM078977 | Chile |
| ZIKV | KM078978 | Chile |
| ZIKV | KM078979 | Chile |
| ZIKV | KJ873160 | New Caledonia |
| ZIKV | KJ873161 | New Caledonia |
| ZIKV | KF993678 | Canada |
| ZIKV | KJ776791 | French Polynesia |
| ZIKV | KF383084 | Senegal |
| ZIKV | KF383085 | Senegal |
| ZIKV | KF383086 | Cote d'Ivoire |
| ZIKV | KF383087 | Senegal |
| ZIKV | KF383088 | Senegal |
| ZIKV | KF383089 | Senegal |
| ZIKV | KF383090 | Senegal |
| ZIKV | KF383091 | Senegal |
| ZIKV | KF383092 | Senegal |
| ZIKV | KF383093 | Senegal |
| ZIKV | KF383094 | Senegal |
| ZIKV | KF383095 | Senegal |
| ZIKV | KF383096 | Senegal |
| ZIKV | KF383097 | Senegal |
| ZIKV | KF383098 | Senegal |
| ZIKV | KF383099 | Senegal |
| ZIKV | KF383100 | Senegal |
| ZIKV | KF383101 | Senegal |
| ZIKV | KF383102 | Senegal |
| ZIKV | KF383103 | Cote d'Ivoire |
| ZIKV | KF383104 | Cote d'Ivoire |
| ZIKV | KF383105 | Cote d'Ivoire |
| ZIKV | KF383106 | Cote d'Ivoire |
| ZIKV | KF383107 | Cote d'Ivoire |
| ZIKV | KF383108 | Cote d'Ivoire |
| ZIKV | KF383109 | Burkina Faso |
| ZIKV | KF383110 | Cote d'Ivoire |
| ZIKV | KF383111 | Cote d'Ivoire |
| ZIKV | KF383112 | Cote d'Ivoire |
| ZIKV | KF383113 | Cote d'Ivoire |
| ZIKV | KF383114 | Senegal |
| ZIKV | KF383115 | Central African Republic |
| ZIKV | KF383116 | Senegal |
| ZIKV | KF383117 | Senegal |
| ZIKV | KF383118 | Senegal |
| ZIKV | KF383119 | Senegal |
| ZIKV | KF268948 | Central African Republic |
| ZIKV | HQ234498 | Uganda |
| ZIKV | HQ234499 | Malaysia |
| ZIKV | HQ234500 | Nigeria |
| ZIKV | HQ234501 | Senegal |
| ZIKV | JN860885 | Cambodia |
| ZIKV | EU545988 | Micronesia |
| ZIKV | KU646827 | Colombia |
| ZIKV | KU646828 | Colombia |
| ZIKV | KU312313 | Suriname |
| ZIKV | KU312314 | Suriname |
| ZIKV | KU312315 | Suriname |
| ZIKV | KJ634273 | Cook Islands |
| ZIKV | KF383015 | Senegal |
| ZIKV | KF383016 | Senegal |
| ZIKV | KF383017 | Senegal |
| ZIKV | KF383018 | Senegal |
| ZIKV | KF383019 | Senegal |
| ZIKV | KF383020 | Cote d'Ivoire |
| ZIKV | KF383021 | Senegal |
| ZIKV | KF383022 | Senegal |
| ZIKV | KF383023 | Senegal |
| ZIKV | KF383024 | Senegal |
| ZIKV | KF383025 | Senegal |
| ZIKV | KF383026 | Senegal |
| ZIKV | KF383027 | Senegal |
| ZIKV | KF383028 | Senegal |
| ZIKV | KF383029 | Senegal |
| ZIKV | KF383030 | Burkina Faso |
| ZIKV | KF383031 | Senegal |
| ZIKV | KF383032 | Senegal |
| ZIKV | KF383033 | Senegal |
| ZIKV | KF383034 | Senegal |
| ZIKV | KF383035 | Uganda |
| ZIKV | KF383036 | Cote d'Ivoire |
| ZIKV | KF383037 | Cote d'Ivoire |
| ZIKV | KF383038 | Cote d'Ivoire |
| ZIKV | KF383039 | Senegal |
| ZIKV | KF383040 | Cote d'Ivoire |
| ZIKV | KF383041 | Cote d'Ivoire |
| ZIKV | KF383042 | Cote d'Ivoire |
| ZIKV | KF383043 | Cote d'Ivoire |
| ZIKV | KF383044 | Cote d'Ivoire |
| ZIKV | KF383045 | Cote d'Ivoire |
| ZIKV | KF383046 | Cote d'Ivoire |
| ZIKV | KF270886 | Gabon |
| ZIKV | KU740184 | China |
| ZIKV | KU720415 | Uganda |
| ZIKV | KU681082 | Philippines |
| ZIKV | KU681081 | Thailand |
| ZIKV | KU497555 | Brazil |
| ZIKV | KU527068 | Brazil |
| ZIKV | KU647676 | Martinique |
| YFV | JF912184 | Brazil |
| YFV | JF912181 | Brazil |
| SPOV | NC_029055 | South Africa |
